# The need to tailor the omission of axillary lymph node dissection to patients with good prognosis and sentinel node micro‐metastases

**DOI:** 10.1002/cam4.5257

**Published:** 2022-09-20

**Authors:** Gilles Houvenaeghel, Alexandre de Nonneville, Nicolas Chopin, Jean‐Marc Classe, Chafika Mazouni, Marie‐Pierre Chauvet, Fabien Reyal, Christine Tunon de Lara, Eva Jouve, Roman Rouzier, Emile Daraï, Pierre Gimbergues, Charles Coutant, Anne Sophie Azuar, Richard Villet, Patrice Crochet, Sandrine Rua, Marie Bannier, Monique Cohen, Jean‐Marie Boher

**Affiliations:** ^1^ Department of Surgical Oncology CRCM, Institut Paoli‐Calmettes, Aix‐Marseille Univ, CNRS, INSERM Marseille France; ^2^ Department of Medical Oncology CRCM, Institut Paoli‐Calmettes, Aix‐Marseille Univ, CNRS, INSERM Marseille France; ^3^ Centre Léon Bérard, 28 rue Laennec Lyon France; ^4^ Institut René Gauducheau Site hospitalier Nord St Herblain France; ^5^ Institut Gustave Roussy Villejuif France; ^6^ Centre Oscar Lambret Lille France; ^7^ Institut Curie Paris France; ^8^ Institut Bergonié Bordeaux France; ^9^ Centre Claudius Regaud Toulouse France; ^10^ Centre René Huguenin Saint Cloud France; ^11^ Hôpital Tenon Paris France; ^12^ Centre Jean Perrin Clermont Ferrand France; ^13^ Centre Georges François Leclerc Dijon France; ^14^ Hôpital de Grasse Grasse France; ^15^ Hôpital des Diaconnesses Paris France; ^16^ Hôpital de la Conception Marseille France; ^17^ Department of Biostatistics and Methodology, Institut Paoli Calmettes, 13009 and Aix‐Marseille University, Unité Mixte de Recherche S1252 Institut de Recherche pour le Développement Marseille France

**Keywords:** adjusted Kaplan–Meier estimator, axillary lymph node dissection, breast cancer, micro‐metastasis, sentinel node

## Abstract

**Background:**

Results of IBCSG‐23‐01‐trial which included breast cancer patients with involved sentinel nodes (SN) by isolated‐tumor‐cells or micro‐metastases supported the non‐inferiority of completion axillary‐lymph‐node‐dissection (cALND) omission. However, current data are considered insufficient to avoid cALND for all patients with SN‐micro‐metastases.

**Methods:**

To investigate the impact of cALND omission on disease‐free‐survival (DFS) and overall survival (OS), we analyzed a cohort of 1421 patients <75 years old with SN‐micro‐metastases who underwent breast conservative surgery (BCS). We used inverse probability of treatment weighting (IPTW) to obtain adjusted Kaplan–Meier estimators representing the experience in the analysis cohort, based on whether all or none had been subject to cALND omission.

**Results:**

Weighted log‐rank tests comparing adjusted Kaplan–Meier survival curves showed significant differences in OS (*p*‐value = 0.002) and borderline significant differences in DFS (*p*‐value = 0.090) between cALND omission versus cALND. Cox's regression using stabilized IPTW evidenced an average increase in the risk of death associated with cALND omission (HR = 2.77, CI95% = 1.36–5.66). Subgroup analyses suggest that the rates of recurrence and death associated with cALND omission increase substantially after a large period of time in the half sample of women less likely to miss cALND.

**Conclusions:**

Using IPTW to estimate the causal treatment effect of cALND in a large retrospective cohort, we concluded cALND omission is associated with an increased risk of recurrence and death in women of <75 years old treated by BCS in the absence of a large consensus in favor of omitting cALND. These results are particularly contributive for patients treated by BCS where cALND omission rates increase over time.

## Graphical abstract

To investigate the impact of completion axillary lymph node dissection (ALND) omission in patients with sentinel node micro‐metastases, we retrospectively analyzed a large cohort of 1421 patients with SN micro‐metastases who underwent breast conservative surgery (BCS) in 15 French academic centers. We used inverse probability of treatment weighting (IPTW) to obtain adjusted Kaplan‐Meier estimators representing the experience in the analysis cohort. Weighted log‐rank test comparing adjusted Kaplan‐Meier survival curve showed significant difference for overall survival according to completion axillary lymph node dissection (cALND) or no cALND.

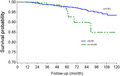

## 1 INTRODUCTION

Omission of completion axillary lymph node dissection (cALND) is recommended for patients with early breast cancer (BC) up to 5 centimeters without axillary adenopathy and negative sentinel nodes (SNs) to avoid adverse effects of ALND since the NSABP B‐32 trial.^1^ Since the ACOSOG Z0011 trial,^2^, ^3^ omission of cALND could also be extended to patients with SNs involved by 1 or 2 micro‐ or macro‐metastases without extracapsular extension, when treated by breast conservative surgery (BCS) followed by whole breast radiotherapy and systemic adjuvant therapy (adjuvant chemotherapy and or endocrine therapy). More recently, results of the IBCSG 23–01 trial^4^, ^5^ which included patients with SNs involvement by either isolated tumor cells (ITC) or micro‐metastases, and treated by BCS or mastectomy, supported the non‐inferiority of cALND omission. However, some limitations to conclude non‐inferiority of cALND omission in comparison with cALND were reported, particularly for patients with SN micro‐metastases and total mastectomy, related to the very low number of patients included in this situation in IBCSG 23–01 trial, and current guidelines consider data as insufficient to avoid cALND as a consensual standard clinical practice for SNs micro‐metastases. In IBCSG 23–01 trial, results were reported for all patients without distinction between SNs ITC or micro‐metastases with very few cases of mastectomy. In, a previous study, we have reported an increased risk of death in case of cALND omission in patients with SNs micro‐metastases.^6^ An ongoing randomized trial (SERC trial)^7^, ^8^ may clarify this issue by assessing the non‐inferiority of cALND omission versus cALND for patients with SNs involved by ITC, micro‐metastases, or macro‐metastases with a planned stratification between macro‐metastases and occult metastases, but survival results are not achieved now. To investigate the impact of cALND omission on disease‐free survival (DFS) and overall survival (OS), we retrospectively analyzed in a larger multicentric cohort of patients with SN‐micro‐metastases who underwent BCS, with longer follow‐up than our previous study,^6^ patients with BCS and SNs micro‐metastases.

## 2 METHODS

### 2.1 Patients

Clinical data were extracted from a multicentric retrospective database comprising 23,134 patients who underwent primary surgery for early BC in 15 different French academic centers (NCT03461172^9^, ^10^, ^11^, ^12^, ^13^, ^14^). Patients <75 years who underwent BCS and sentinel lymph node biopsy (SLNB) without neoadjuvant therapy, with SN micro‐metastases involvement (>0.2 mm to <=2 mm) confirmed by pathologic examination, with or without cALND were included. Exclusion criteria were the absence of SLNB; patients with SN macro‐metastases, pN0(i+)sn, pN0sn, SLNB, or ALND after NAC; SN micro‐metastasis lost of follow‐up; patients receiving mastectomy or with unknown type of surgery; Neo adjuvant chemotherapy; or age ≥ 75 years. SLNB was performed for patients with invasive BC < = 5 cm, axillary cN0, detected by combined isotopic and colorimetric method or only isotopic detection, with peri‐tumoral or retro areolar injection. Pathologic examination included immunohistochemically analysis in case of a negative result on standard hematoxylin and eosin analysis on serial sections.^15^ Positive endocrine receptor (ER) status and HER2 status were determined according to French guidelines (estrogen receptor and/or progesterone receptor by immunochemistry [IHC] with a 10% threshold for ER positivity; IHC HER2 3+ and/or HER2 amplification by in situ hybridization). Adjuvant chemotherapy (AC) was administered according to guidelines used in each center and whole breast radiotherapy (WBR) was systematically performed after BCS. Regional node irradiation was not systematically realized.

### 2.2 Study design and statistics

The primary objective was to assess the causal effect of cALND omission on DFS and OS in patients with BC and SNs micro‐metastases, defined as the comparisons of the potential outcomes for the same individual who received two different treatments.^16^ Main prognostic factors assessed before surgery and treatment characteristics were first summarized using standard descriptive statistics in the experimental group (cALND omission) and the control group (cALND) and compared using the Chi‐square test to identify potential confounding variables that affect both the treatment and the outcomes. The outcome variables were first summarized using naïve methods (Kaplan–Meier and log‐rank tests) in women undergoing BCS. To minimize the effects of observable confounding,^17^ we used inverse probability of treatment weights (IPTW) to estimate adjusted Kaplan–Meier survival curves (AKM) representing the experience in the analysis set, based on whether all or none had been subject to the omission of cALND.

Using multivariate logistic regression, we first derived individual propensity score $\mathrm{ps}\left(X\right)$ defined as the conditional probability of cALND omission given a set of potential confounders X. The multidimensional vector X included all prognostic factors and adjuvant treatment characteristics with significant or borderline significant univariate association with cALND omission in the analysis cohort (*p*‐values <= 0.20). To adequately control the type I error rate, we used stabilized individual weights (sIPTW), defined as the inverse of the conditional probability of receiving the actual treatment (IPTW) multiplied by the proportion of patients in the same sub‐cohort who received the same treatment.

$$w=\frac{Z}{\mathrm{ps}\left(X\right)}+\frac{1-Z}{1-\mathrm{ps}\left(X\right)}$$

The aim is to achieve a balance in observable potential confounding variables between treated (cALND) and non‐treated (no cALND) patients in a sample weighted by the inverse probability of treatment. We used standardized differences in proportions to assess the balance achieved of important prognostic risk and adjuvant therapies factors at each level category and consider a standardized difference in absolute value below 10% as indicative of negligible imbalance.^18^ Weighted Cox proportional hazards regression (sIPTW‐Cox) analysis with robust sandwich variance estimate was used to estimate the magnitude of the causal effect of cALND omission on DFS and OS. The same analyses were conducted to evaluate the causal effect of cALND omission in subgroups of patients associated with low or high propensity scores (below and above the sample medians). Statistical analyses were carried out using SAS release 9.4 (SAS‐Institute, Inc., Cary, NC). This study adheres to the STROBE standard for observational study.

## 3 RESULTS

### 3.1 Population characteristics

We included 1451 patients treated between 1999 and 2012 at 15 sites for early BC by BCS and SNs micro‐metastases (Table 1, Figure S1). Of the 1266 patients who underwent cALND (87.3%), the pN status final was unknown in 212 patients and macro‐metastases at cALND were found in 112 patients (10.6%). Adjuvant chemotherapy was more often prescribed in the cALND group (57.5% vs. 45.4%; *p* = 0.002). The median ages for patients without cALND and with cALND were 58.5 years (range 36–74) and 55.0 years (range 25–74), respectively. The median duration of follow‐up was 70.0 months (CI 95% 68.0–71.5) for the global cohort; 65.4 months (CI 95% 62–66) for the 185 patients without cALND (61.7% at 5 years, CI 95% 54.2–68.3 and 14.2% at 7 years, CI 95% 9.5–19.9); and 71.2 months (CI 95% 69.1–73.0) for the 1266 patients with cALND (66.2% at 5 years, CI 95% 63.5–68.7 and 34.9% at 7 years, CI 95% 32.2–37.6). Omission of cALND was more frequent in patients treated during the second period (after 2004) (9.1% vs. 3.8%: *p* < 0.0001).

**TABLE 1:** Characteristics of patients according to completion axillary lymph node dissection (cALND) or no cALND

		cALND (*N* = 1266)		No cALND (*N* = 185)		Wilcoxson or Khi2
		Nb	%	Nb	%	*p*‐value
Age	≤ 50	428	33.8	49	26.5	**0.047**
	51–74	837	66.2	136	73.5	
	Missing	1				
Number SN	≤ 2	835	66.0	99	55.5	**0.001**
	> 2	431	34.0	86	46.5	
Number positive‐SN	≤ 2	1259	99.5	184	99.5	0.983
	> 2	7	0.6	1	0.5	
Tumor size	≤ 20 mm	1021	81.5	156	84.8	0.278
	> 20 mm	232	18.5	28	15.2	
	Missing	13		1		
Histology	Ductal	1063	84.0	148	80.0	0.254
	Lobular	108	8.5	17	9.2	
	Mixed/others	94	7.4	20	10.8	
	Missing	1				
Grade SBR	1	464	37.2	72	39.8	0.709
	2	585	46.9	79	43.6	
	3	198	15.9	30	16.6	
	Missing	19		4		
LVI	No	830	70.9	136	77.7	0.061
	Yes	341	29.1	39	22.3	
	Missing	95		10		
Endocrine receptors	Negative	89	7.1	10	5.5	0.431
	Positive	1162	92.9	171	94.5	
	Missing	15		4		
Her2 status	Negative	699	93.1	139	96.5	0.120
	Positive	52	6.9	5	3.5	
	missing	515		41		
pN status final	Micro	942	89.4	185	100	**<0.001**
	Macro	112	10.6			
	Missing	212				
Chemotherapy	No	508	42.5	101	54.5	**0.002**
	Adjuvant	688	57.5	84	45.4	
	Missing	70				
Radiotherapy	No	32	2.8	9	4.9	0.117
	Yes	1123	97.2	174	95.1	
	Missing	111		2		
Regional Nodes Irradiation	No	461	44.4	97	57.4	**0.002**
	Yes	577	55.6	72	42.6	
	Missing	228		16		
Endocrine therapy	No	122	10.2	23	12.4	0.353
	Yes	1076	89.8	162	87.6	
	Missing	68				
Periods	1999–2004	587	46.4	56	30.3	**<0.001**
	> 2004	679	53.6	129	69.7	

Abbreviations: LVI, lympho‐vascular invasion; SN, sentinel node.

Value in bold correspond to statiscticaly significant *p*‐values.

### 3.2 Propensity score model and stabilized inverse probability of treatment weight estimation

Multivariable logistic regression was used to derive individual propensity scores in the analysis cohort. Factors with borderline significant evidence of univariate association with cALND omission (*p* ≤ 0.25) were used as regression variables to predict the conditional probability of cALND omission. Due to high numbers of missing values, HER2 status who met this criterion was excluded from the list of regressors. Odd ratios estimated with 95% CIs and p‐values are reported in Table 2. The lack of regional irradiation, more than two sentinel nodes collected, and entry date into the study beyond 2005 were the only independent factors identified as positively correlated with the omission of cALND in the multivariate analysis. The median propensity score was 0.13 (range 0.05–0.30). There is a high degree of overlap of the distribution of propensity scores between study groups (Figure S2). Nomograms summarizing the contribution of each factor included in the model prediction are depicted in Figure S3. Based on the absolute values of the standardized difference (Table S1), a strong imbalance between key characteristics was observed for final lymph node invasion status. Other moderate imbalances were observed for the histology subtype, the SBR grade, and HER2 status. All patients with macometastases underwent lymph node dissection, of whom 66 patients with a propensity score below the median score. Comparison of Kaplan–Meier curves showed a highly significant negative impact on disease‐free survival (*p* = 0.003) and overall survival (*p* = 0.006).

**TABLE 2:** Odd ratios estimates with 95% CI from logistic regression models predicting cALND omission

Multivariate analysis	Parameter	Odds Ratio [95% CI]	*p*‐value
Age in class	≤ 50 vs. 51–74	0.76 [0.51, 1.12]	0.165
Chemotherapy	Adjuvant vs. No	0.81 [0.56, 1.19]	0.281
LVI	Yes vs. No	0.78 [0.52, 1.18]	0.243
RNI	Yes vs. No	0.68 [0.47, 0.97]	0.036
Number SN Harvested	>2 vs. ≤2	1.71 [1.21, 2.42]	0.002
Period	≥ 2005 vs. < 2005	1.58 [1.08, 2.32]	0.020

Abbreviations: LVI, lympho‐vascular invasion; RNI, regional node irradiation; SN, sentinel node.

### 3.3 Overall survival for patients with or without cALND

**FIGURE 1:**
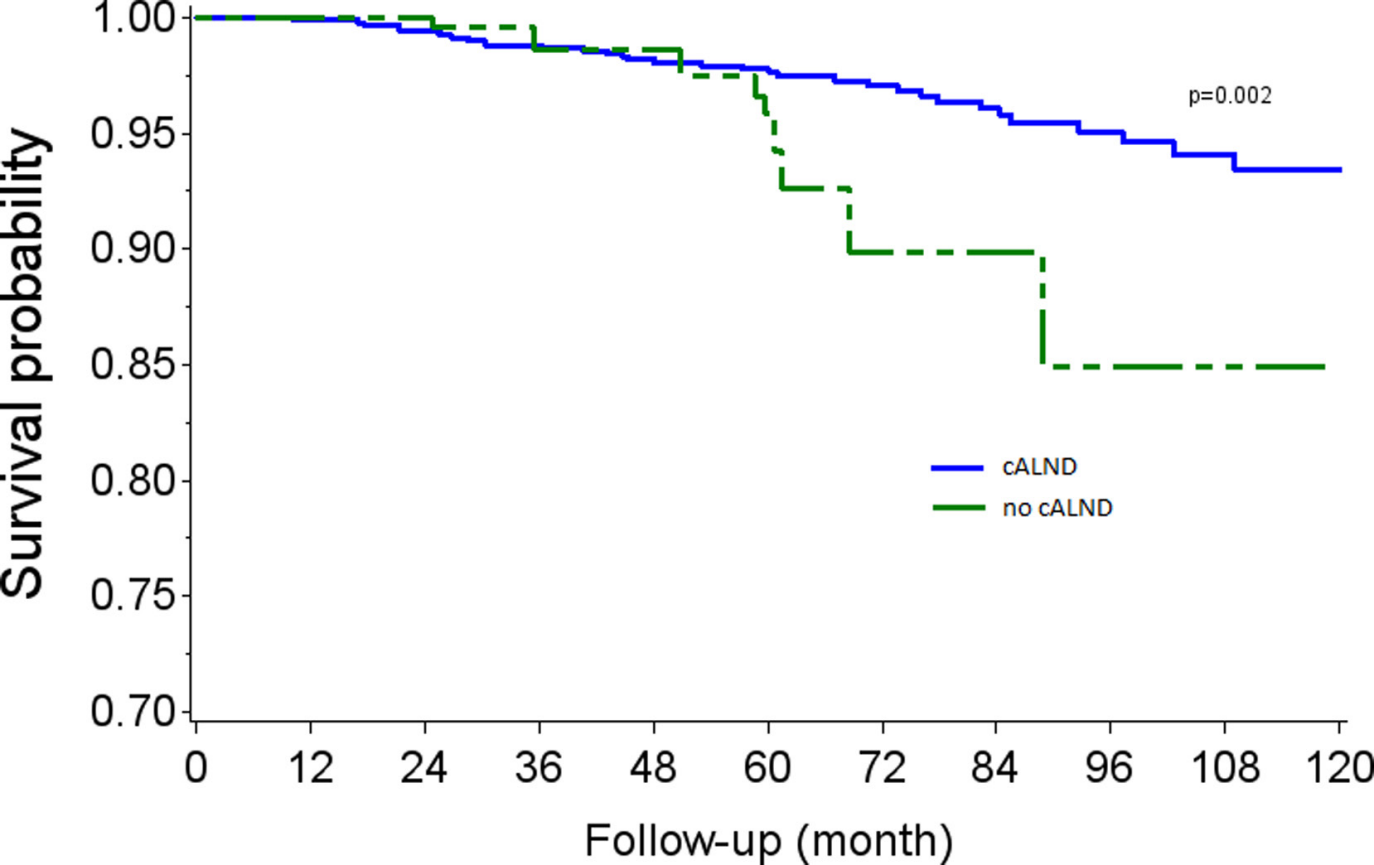
Adjusted Kaplan–Meier estimates for overall survival according to completion axillary lymph node dissection (cALND) or no cALND

OS rates at 5 and 7 years were, respectively, 97.3% (CI 95% 96.1–98.1) and 95.5% (CI 95% 93.9–96.7) in the cALND group, and 96.4% (CI 95% 91.6–98.5) and 87.3 (CI 95% 74.5–93.9) in the group without cALND (*p* = 0.023, HR: 2.05, CI 95% 1.09–3.86). In univariate analysis, OS rates were significantly associated with Grade 2 (*p* = 0.0292), Grade 3 (*p* < 0.0001), positive endocrine receptors (*p* < 0.0001), and presence of lymphovascular infiltration (*p* = 0.0340). Tumor size >20 mm was of borderline significance (*p* = 0.0625). Adjusted Kaplan–Meier estimates representing the experience, based on whether all or none had been subject to the omission of cALND in the analysis cohort are reported in Figure 1. Weighted Log‐rank test comparing AKM survival curves detected a significant association between the omission of cALND versus cALND (p‐value = 0.002). The main differences were observed after some period of time, where survival curves estimators diverged leading to better survival rates with cALND. Cox's regression using stabilized IPTW evidenced an average increase in the risk of death associated with cALND omission (HR: 2.77, CI95% 1.36–5.66).

### 3.4 Disease‐free survival with or without cALND

**FIGURE 2:**
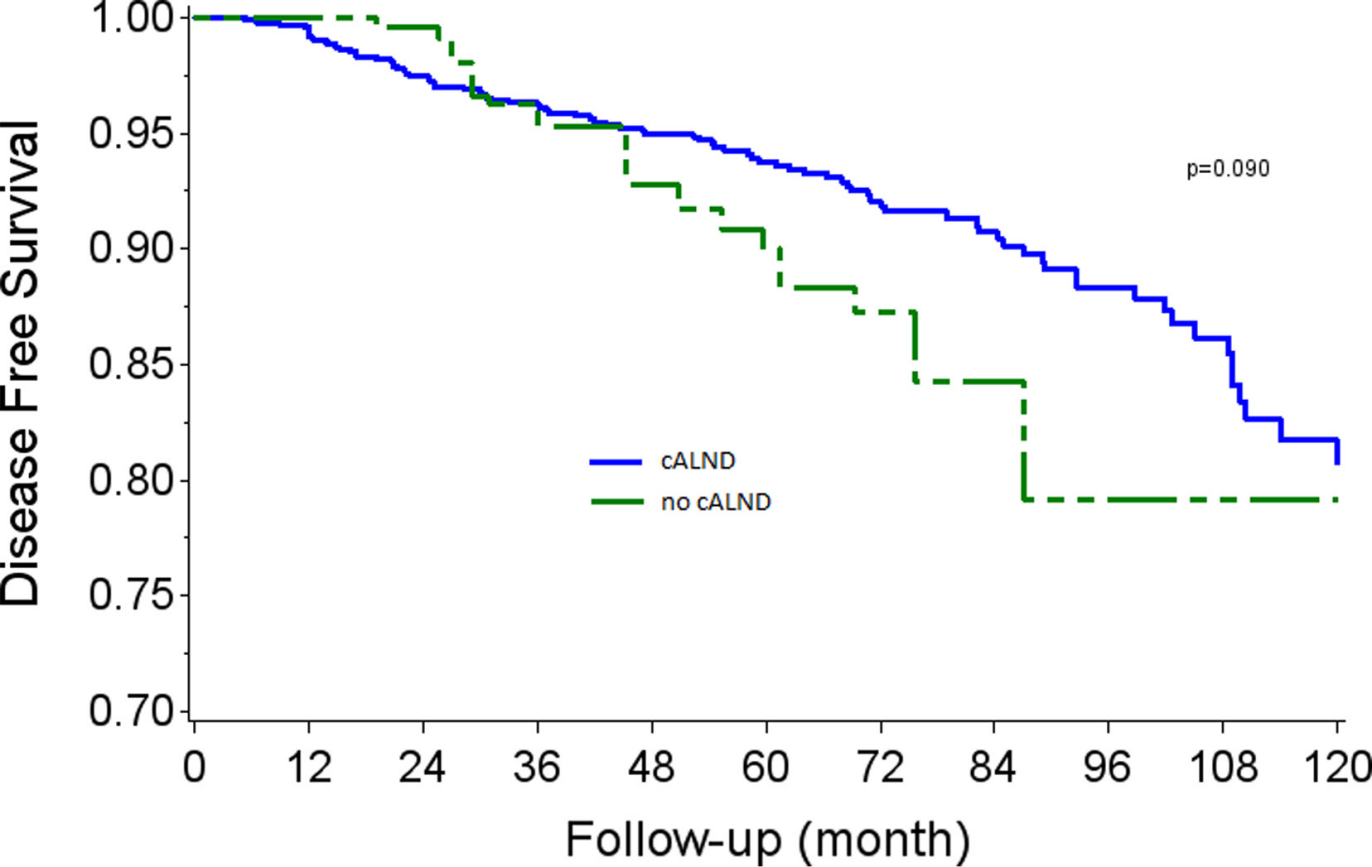
Adjusted Kaplan–Meier estimates for disease‐free survival according to completion axillary lymph node dissection (cALND) or no cALND

DFS rates at 5 and 7 years were, respectively, 93.2% (CI 95% 91.5–94.5) and 90.5% (CI 95% 88.3–92.2) in the cALND group, and 90.9% (CI 95% 85.0–94.5) and 84.7% (CI 95% 75.0–90.9) in the cALND omission group (*p* = 0.143, HR: 1.42 CI 95% 0.88–2.29). In univariate analysis, DFS rates were significantly associated with tumor size >20 mm (*p* < 0.001), Grade 2 (*p* = 0.0065), Grade 3 (*p* < 0.0001), presence of LVI (*p* = 0.0043), positive endocrine receptors (*p* = 0.0023). AKM survival estimates according to whether all or none had been subject to the omission of cALND in each sub‐cohort are reported in Figure 2. Weighted Log‐rank test comparing AKME survival curves detected a borderline significant association between the omission of cALND versus cALND (p‐value = 0.090, HR: 1.58 CI 95% 0.91–2.74).

### 3.5 Subgroup analysis in BCS sub‐cohort

**FIGURE 3:**
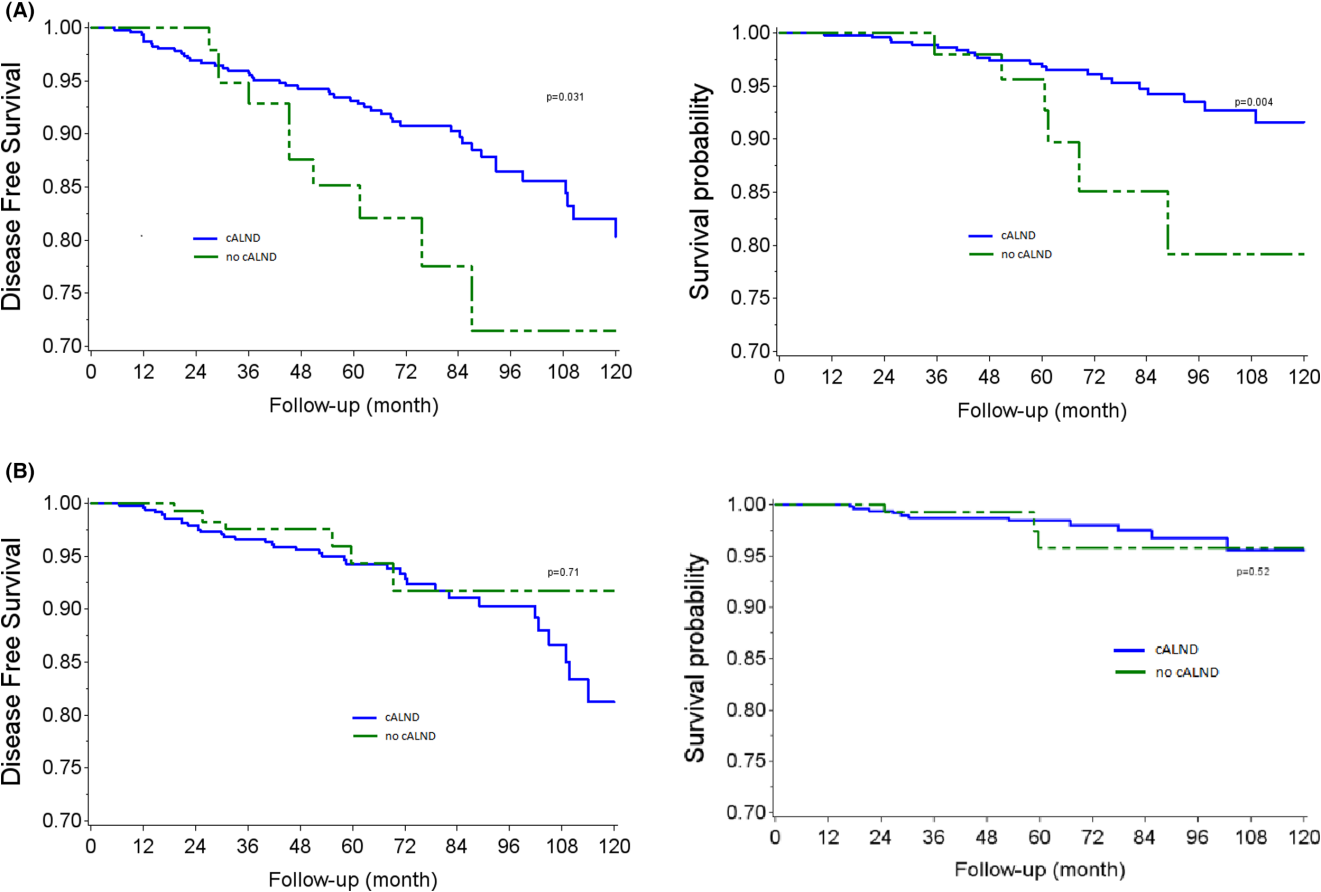
(A) Adjusted Kaplan–Meier estimates for DFS and OS according to completion axillary lymph node dissection (cALND) or no cALND: BCS sub‐cohort with ps < = 0.13. (B) Adjusted Kaplan–Meier estimates for DFS and OS according to completion axillary lymph node dissection (cALND) or no cALND: BCS sub‐cohort with ps > 0.13.

We evaluated the causal effect of cALND omission on OS and DFS in two specific subgroups (Table S2): patients with low propensity scores (*sub‐cohort with ps ≤ 0.13*) or high propensity scores (*sub‐cohort with ps > 0.13*). AKM survival estimates according to whether all or none had been subject to cALND in these specific subgroups are reported in Figure 3B. Weighted Log‐rank tests detected significant differences in DFS (*p*‐value = 0.031) and OS (*p*‐value = 0.004) in the half sample of women less likely to miss cALND (*ps < =0.13*). Average hazard ratios for DFS and OS were, respectively, 2.09 (CI 95% 1.06–4.14) and 3.23 (CI 95% 1.41–7.40) in the *sub‐cohort with ps < = 0.13 and 0.84* (CI 95% 0.35–2.03) and 1.53 (CI 95% 0.43–5.41) in the *sub‐cohort with ps > 0.13*.

## 4 DISCUSSION

The omission of ALND completion in patients with SN ITC or micro‐metastases did not show a negative impact on RFS in the IBCSG 23–01 trial^4^, ^5^ and in our previous study.^6^ However, there was no distinction between ITC and micro‐metastases in the IBCSG 23–01 trial that included 643 patients with SN tumor size ≤1 mm (69.1%), 266 patients with SN tumor size between 1.1 and 2 mm (28.6%), and 21 patients with SN tumor size >2 mm (2.3%). In our previous study,^6^ we reported 1390 patients with SN micro‐metastases (69.2%) and 619 patients with SN ITC. Moreover, in IBCSG 23–01 trial,^4^, ^5^ 181 patients were treated with intraoperative radiotherapy only (*n* = 159) or no radiotherapy (*n* = 22). In our previous study^6^ mastectomy rate was 10.0% (181 among 1800 patients documented). In the present study, only SN micro‐metastases with or without cALND for patients <=75 years old who underwent BCS, without neoadjuvant treatment were included. Patients treated by mastectomy (n = 260) were excluded due to a low number of patients and a very low number of events. Patients who were 75 years old or more were excluded due to a low number of patients with cALND in this population with a good prognosis and associated with a low level of propensity score. However, results observed with patients ≥75 years old do not modify the reported conclusions.

cALND was significantly associated with several criteria including the period of treatment with higher rate of cALND omission during the second period (after 2004). A higher rate of AC was reported for patients with cALND (57.5%) in comparison with patients without cALND (45.4%) (Table 1). The rate of invaded non‐sentinel nodes (NSN) with one or several macro‐metastases at cALND was 10.6%. This rate is comparable to previously reported levels for SN micro‐metastases: 10.3% for SN micro‐metastases (22/214) in SERC trial for the first 1855 patients randomized with rates of 4.4% (4/92) without chemotherapy, 6.9% (2/29) with chemotherapy administered before cALND and 17.7% (15/85) with chemotherapy administered after cALND for SN micro‐metastases. In the study published by Tvedskov et al,^19^ the rates of involved NSN were 9.2% for SN ITC and 17.9% for SN micro‐metastases, and in a previous study we had reported positive‐NSN rates of 13.9% (40/287) for ITC and 14.1% (93/658) for pN1mi SN with a predictive nomogram based on tumor size, ratio of positive‐SN number/analyzed‐SN number, LVI, tumor histology.^20^, ^21^

Weighted log‐rank tests comparing AKM survival curves detected significant results on OS, with a significant negative impact of cALND omission. However, only significant associations for OS and DFS were observed in half the sample of women treated by BCS who are less likely to miss cALND. Among this subgroup (level ≤0.13), the rates of systemic adjuvant therapy, regional node irradiation, age < =50 years, and presence of LVI (80.3%, 77.5%, 46.3%, and 43.7%, respectively) were higher than the rates observed for patients with ps >0.13. Diagnostics using standardized differences showed similar distributions in observable confounding factors between the treated and non‐treated groups in the sample of patients weighted by the inverse probability of treatment. These results on DFS are concordant with previous results to consider the non‐inferiority of cALND omission for patients with SN micro‐metastases treated by BCS or mastectomy with or without PMRT.^22^

The limitations of this study rely on the retrospective design and the presence of missing data in important confounding variables (as HER2). Of the documented 1451 patients with breast cancer surgery, 349 (24%) were excluded from IPTW analysis due to missing data in the propensity score model. This approach, with direct comparison of weighted Kaplan–Meier survival curves is subject to less bias and more robustness than competing methods based on matched samples. Additional strengths of our study include the large sample size of BCS‐only patients without neoadjuvant treatment; the multicentric recruitment limiting biases inherent in single‐center studies while also reflecting real‐world practice, and the distinction between ITC and micro‐metastases. A high evidence level of non‐inferiority is required to confirm this omission in current practice, for all patients, with new results of randomized trials and or meta‐analysis including patients treated by BCS or mastectomy. In the SERC trial,^7^, ^8^ with an external validation for SN micro‐metastases,^23^ 2207 patients with invaded SNs, treated by BCS or mastectomy, have been randomized between cALND and no ALND with stratification according to the SN tumor size, macro‐metastases or occult metastases: longer follow‐up is necessary to observe sufficient event numbers to present accurate results. Other trials with cALND randomization are in process for SN macro‐metastases: INSEMA trial with only 1 or 2 SN macro‐metastases and conservative treatment,^24^ POSNOC trial with only 1 or 2 SN macro‐metastases and conservative treatment or mastectomy with cALND or radiotherapy versus no other axillary treatment,^25^ SENOMAC trial for patients with 1 or 2 SN macro‐metastases including mastectomies,^26^ and SINODAR trial with 1 or 2 SN macro‐metastases.^27^ BOOG 2013–07 trial, was dedicated to mastectomy.^28^ Unfortunately, the BOOG trial had to be shut down due to insufficient inclusion. SENOMIC trial^29^ was dedicated to patients with SN micro‐metastasis, treated by BCS or mastectomy, without cALND. Axillary radiotherapy could be performed for patients without cALND, but AMAROS trial failed to demonstrate non‐inferiority of axillary radiotherapy in comparison to cALND due to an insufficient number of patients^30^ as reported by authors, and the aim for these patients is to avoid axillary dedicated treatment and morbidity of this therapy.

## 5 CONCLUSION

Using the inverse probability of treatment weighting to estimate the causal treatment effect of cALND in a large retrospective cohort, we concluded that cALND omission is associated with an increased risk of recurrence and death in half of the women treated by breast conservative surgery. In our study setting, where the proportional hazards assumption was violated, direct comparison of weighted Kaplan–Meier survival curves is subject to less bias and more robustness than competing methods based on matched samples. These results are particularly contributive for patients treated by breast conservative surgery where cALND omission rates increase over time in the absence of a large consensus in favor of omitting cALND, potentially leading to under‐staging of patients eligible to systemic therapies.

## AUTHOR CONTRIBUTIONS

**Gilles Houvenaeghel:** Conceptualization (lead); formal analysis (equal); investigation (equal); methodology (equal); resources (equal); supervision (lead); validation (lead); writing – original draft (lead); writing – review and editing (equal). **Alexandre de Nonneville:** Investigation (equal); methodology (equal); resources (equal); visualization (equal); writing – original draft (lead); writing – review and editing (equal). **Nicolas Chopin:** Resources (equal); writing – review and editing (equal). **Jean‐Marc Classe:** Resources (equal); writing – review and editing (equal). **Chafika Mazouni:** Resources (equal); writing – review and editing (equal). **Marie‐Pierre Chauvet:** Resources (equal); writing – review and editing (equal). **Fabien Reyal:** Resources (equal); writing – review and editing (equal). **Christine Tunon de Lara:** Resources (equal); writing – review and editing (equal). **Eva Jouve:** Resources (equal); writing – review and editing (equal). **Roman Rouzier:** Resources (equal); writing – review and editing (equal). **Emile Darai:** Resources (equal); writing – review and editing (equal). **Pierre Gimbergues:** Resources (equal); writing – review and editing (equal). **Charles Coutant:** Resources (equal); writing – review and editing (equal). **Anne Sophie Azuar:** Resources (equal); writing – review and editing (equal). **Richard Villet:** Resources (equal); writing – review and editing (equal). **Patrice Crochet:** Resources (equal); writing – review and editing (equal). **Sandrine Rua:** Resources (equal); writing – review and editing (equal). **Marie Bannier:** Resources (equal); writing – review and editing (equal). **Monique Cohen:** Resources (equal); writing – original draft (equal); writing – review and editing (equal). **Jean‐Marie Boher:** Conceptualization (equal); data curation (equal); formal analysis (equal); investigation (equal); methodology (lead); project administration (equal); supervision (equal); validation (equal); visualization (equal); writing – original draft (equal); writing – review and editing (equal).

## FUNDING INFORMATION

Not applicable.

## CONFLICT OF INTEREST

The authors declare that the research was conducted in the absence of any commercial or financial relationships that could be construed as a potential conflict of interest.

## ETHICS STATEMENT

All procedures performed in this study involving human participants were done in accordance with the French ethical standards and with the 2008 Helsinki declaration. As this was a retrospective non‐interventional study, no formal personal consent was required. An authorization to use the database was obtained from the Paoli‐Calmettes Institute institutional review board (ClinicalTrials.gov NCT03461172).

## Supporting information


Figure S1
Figure S2
Figure S3

## Data Availability

Data are available upon reasonable request. Requests to access these datasets should be directed to houvenaeghelg@ipc.unicancer.fr.
