# Supplementary material for: The need to tailor the omission of axillary lymph node dissection to patients with good prognosis and sentinel node micro‐metastases
Source: Cancer Med. 2022 Sep 20;12(4):4023–32. doi: 10.1002/cam4.5257 (PMC9972015; doi:10.1002/cam4.5257)
Supplement: Supplementary file 1 — Figure S1 Figure S2 Figure S3 [file CAM4-12-4023-s001.docx]

Supplementary Material

**Figure S1:** Flow chart.

**Figure S2:** Frequency distributions of propensity scores at evenly spaced intervals


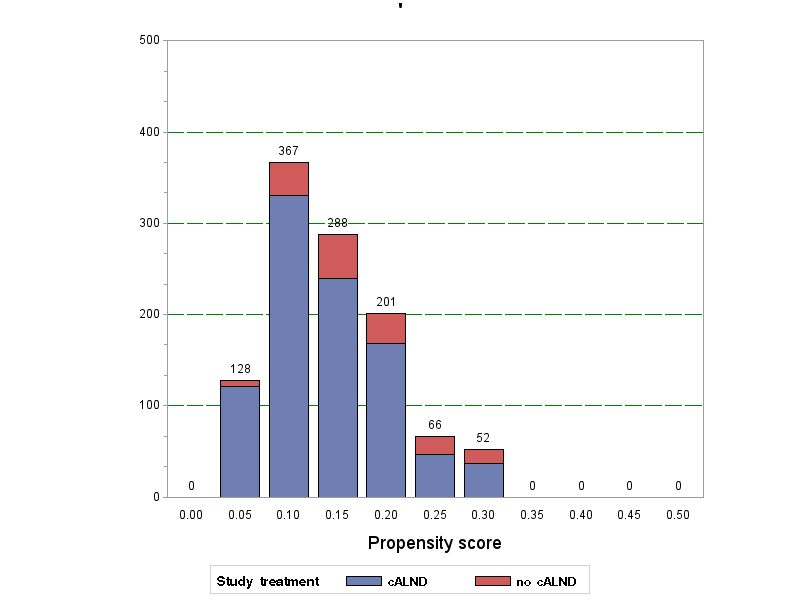


**Figure S3:** Nomogram predicting the conditional probability of no completion of axillary lymph node dissection (cALND) in women of <75 years old undergoing


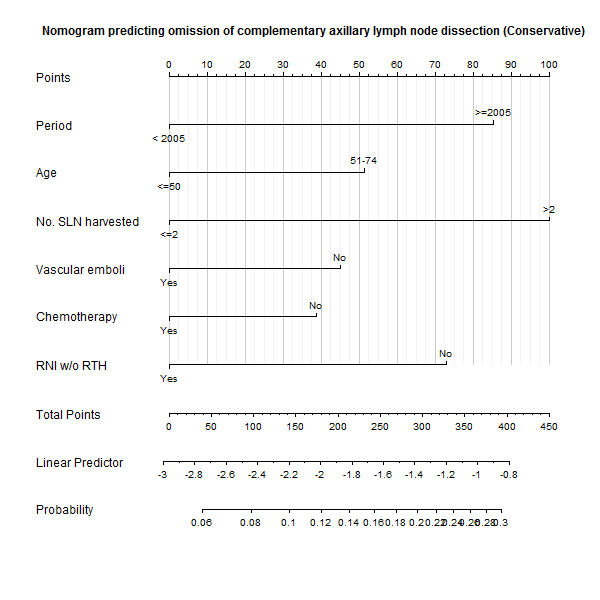


Legend: SLN: sentinel lymph node, RNI: regional nodal irradiation.

**Table S1.** : Characteristics of patients according to completion axillary lymph node dissection (cALND) or no cALND after propensity score weighting.

|  |  | cALND (N=943) | No cALND (N=159) | cALND vs No cALND | |
| --- | --- | --- | --- | --- | --- |
|  |  | % | % | P-value | Standardized difference (%) |
| Age | ≤ 50 | 32.0 | 30.8 | 0.768 | 2.5 |
|  | 51-74 | 68.0 | 69.2 |  | -2.5 |
| Number SN | ≤ 2 | 64.6 | 62.7 | 0.645 | 2.0 |
|  | > 2 | 35.4 | 37.3 |  | -2.0 |
| Number positive-SN | ≤ 2 | 99.3 | 99.2 | 0.812 | 3.9 |
|  | > 2 | 0.7 | 0.8 |  | -3.9 |
| Tumor size | ≤ 20mm | 81.7 | 83.4 | 0.616 | -4.4 |
|  | > 20mm | 18.3 | 16.6 |  | 4.4 |
| Histology | Ductal | 84.5 | 80.1 | 0.187 | **11.7** |
|  | Lobular | 8.3 | 8.5 |  | -1.0 |
|  | Mixed/others | 7.2 | 11.4 |  | **-14.4** |
| Grade SBR | 1 | 39.1 | 32.9 | 0.154 | **13.0** |
|  | 2 | 45.4 | 46;26 |  | -1.8 |
|  | 3 | 15.5 | 20.8 |  | **-13.9** |
| LVI | No | 70.4 | 69.2 | 0.762 | 2.6 |
|  | Yes | 29.6 | 30.8 |  | -2.6 |
| Endocrine receptors | Negative | 6.9 | 5.4 | 0.471 | 6.4 |
|  | Positive | 93.1 | 94.6 |  | -6.4 |
| Her2 status | Negative | 92.9 | 95.7 | 0.289 | **-11.7** |
|  | Positive | 7.1 | 4.3 |  | **11.7** |
| pN status final | Micro | 89.5 | 100 | **<0.001** | **-48.5** |
|  | Macro | 10.5 |  |  | **48.5** |
|  | missing |  |  |  |  |
| Chemotherapy | No | 43.6 | 44.3 | 0.873 | -1.4 |
|  | Adjuvant | 56.4 | 55.7 |  | 1.4 |
|  | missing |  |  |  |  |
| Radiotherapy | No | 3.0 | 3.4 | 0.785 | -2.3 |
|  | Yes | 97.0 | 96.6 |  | 2.3 |
|  | missing |  |  |  |  |
| Regional Nodes Irradiation | No | 44.4 | 45.9 | 0.724 | -3.0 |
|  | Yes | 55.6 | 54.1 |  | 3.0 |
|  | missing |  |  |  |  |
| Endocrine therapy | No | 10.6 | 10.8 | 0.941 | -0.6 |
|  | Yes | 89.4 | 89.2 |  | 0.6 |
|  | Missing |  |  |  |  |
| Periods | 1999-2004 | 38.6 | 38.0 | 0.883 | 1.26 |
|  | > 2004 | 61.4 | 62.0 |  | -1.26 |
